# Transcriptomics and Metabolite Analysis Reveals the Molecular Mechanism of Anthocyanin Biosynthesis Branch Pathway in Different *Senecio cruentus* Cultivars

**DOI:** 10.3389/fpls.2016.01307

**Published:** 2016-09-05

**Authors:** Xuehua Jin, He Huang, Lu Wang, Yi Sun, Silan Dai

**Affiliations:** ^1^College of Landscape Architecture, Beijing Forestry UniversityBeijing, China; ^2^Faculty of Architecture and City Planning, Kunming University of Science and TechnologyKunming, China

**Keywords:** *Senecio cruentus*, colour pigmentation, cyanidin, delphinidin, pelargonidin

## Abstract

The cyanidin (Cy), pelargonidin (Pg), and delphinidin (Dp) pathways are the three major branching anthocyanin biosynthesis pathways that regulate flavonoid metabolic flux and are responsible for red, orange, and blue flower colors, respectively. Different species have evolved to develop multiple regulation mechanisms that form the branched pathways. In the current study, five *Senecio cruentus* cultivars with different colors were investigated. We found that the white and yellow cultivars do not accumulate anthocyanin and that the blue, pink, and carmine cultivars mainly accumulate Dp, Pg, and Cy in differing densities. Subsequent transcriptome analysis determined that there were 43 unigenes encoding anthocyanin biosynthesis genes in the blue cultivar. We also combined chemical and transcriptomic analyses to investigate the major metabolic pathways that are related to the observed differences in flower pigmentation in the series of *S. cruentus*. The results showed that mutations of the *ScbHLH17* and *ScCHI1/2* coding regions abolish anthocyanin formation in the white and the yellow cultivars; the competition of the *ScF3′H1, ScF3′5′H*, and *ScDFR1/2* genes for naringenin determines the differences in branching metabolic flux of the Cy, Dp, and Pg pathways. Our findings provide new insights into the regulation of anthocyanin branching and also supplement gene resources (including *ScF3′5 ′H*, *ScF3′H*, and *ScDFRs*) for flower color modification of ornamentals.

## Introduction

Flower color produces some of the most beautiful displays in nature and serves an important function in the ecology and evolution of plants by attracting animal pollinators (Glover, 2007). Flower color falls into three classes of pigment: flavonoids, carotenoids and betalains, among which anthocyanins, a colored class of flavonoids, confer color diversity ranging from orange and red to violet and blue. Carotenoids and betalains generally yield yellow or red colors.

In the anthocyanin biosynthesis pathway (ABP), the three main branches, Cy, Dp and Pg, are known to differ in the hydroxylation pattern of the B-ring. Pg derivatives provide the basis for orange-red hues (one hydroxyl group), Cy derivatives for deep red hues (two hydroxyl groups) and Dp derivatives for blue hues (three hydroxyl groups; Boase et al., 2010). These three types of anthocyanidin production are quite variable, as stated by Wessinger and Rausher (2012). Specifically, they report that 20% of the species produce Pg, 44% produce Cy, and 45% produce Dp (Wessinger and Rausher, 2012). The metabolism of the three branches of anthocyanin determines the diversity of flower colors. An understanding of the mechanism that regulates the three branches is very important for evolution, agricultural production and genetically modified breeding. From a commercial point of view, the top-selling cut flower species, including *Rosa hybrida* (rose), *Chrysanthemum morifolium* (chrysanthemum), *Dianthus caryophyllus* (carnation) and *Lilium* spp. (lily), occupy more than 50% of the cut-flower market (Tanaka et al., 2008). These flowers can only naturally accumulate Cy and lack the blue flower color series. An understanding of the regulation of anthocyanin branching and an investigation of the key genes underlying this process will be important for the reconstruction of new anthocyanin branches and for the modification of flower colors in these ornamental plants.

Flux changes down these three different branches of the ABP might result from one of the following reasons: loss of function or reduced expression of the genes coding for the branching enzymes, alteration of the substrate specificity due to a mutation in the gene for dihydroflavonol 4-reductase (DFR), or anthocyanidin synthase (ANS) making the enzyme unable to metabolize the specific precursor (Hopkins and Rausher, 2011). Meanwhile, most of the key ABP genes were encoded by gene families, each of which is considered to be derived from gene duplication events and subsequent positive selection (Des Marais and Rausher, 2008). The different copies have evolved either to function in different tissues or at different times or to specialize in their use of different but related substrates (Des Marais and Rausher, 2008; Martins et al., 2013). The way in which flux is controlled in the branched flavonoid pathway has remained largely unknown. It is therefore important to discover the key genes controlling the branching of the pathway.

Each species usually accumulates limited types of anthocyanins and exhibits specific types of flower color. Most plants could only accumulate one or two types of anthcyanins. *Senecio cruentus* is a member of the Compositea family of plants. *S. cruentus* is rich in flower color and in its forms of landscape application, specifically, it can be used for both potted and outdoor landscaping. Most importantly, in *S. cruentus*, many closely related cultivars display dramatically diverse floral color patterns and can synthesize all three of the anthocyanin branches, providing an outstanding study system to understand the molecular principles underlying phenotypic diversification, plant–pollinator interactions and the mechanism regulating anthocyanin branching.

Currently, the increased ease and efficiency of RNA sequencing (RNA-Seq) facilitates the study of the mechanisms underlying metabolite variation for non-model plants, such as *Phalaenopsis* orchids (Hsu et al., 2015), Asiatic hybrid lilies (*Lilium* spp.; Li et al., 2015), *Mimulus* spp. (Yuan et al., 2014), and chrysanthemum (Hong et al., 2015). Furthermore, the combination of the metabolomics and transcriptomics results could allow us to gain more insight into the metabolic flow and mechanism of regulation. For example, using a combination of chemical analysis and RNA-Seq technology, scientists have deduced the major metabolic pathways of *Muscari* flower pigmentation and have examined the candidate genes responsible for the loss of pigmentation (Lou et al., 2014). In *Dahlia pinnata* (dahlia), through both pigment and molecular analyses, black and purple cultivars have been compared. These findings demonstrate that the black dahlia cultivar accumulated a high amount of total anthocyanidins without flavone accumulation, which was determined by the low expression of the *flavone synthase* (*FNS*) gene (Deguchi et al., 2013).

In this study, we constructed the first gene library of *S. cruentus* by using RNA-seq approach. Combining chemical analysis and transcriptomic analysis, the major metabolic pathways underlying flower pigmentation of the different series of *S. cruentus* were investigated and the candidate genes that determine the biosynthesis of anthocyanin and its branching formation were isolated.

## Materials and Methods

### Plant Material and Definition of Flower Developmental Stages

**FIGURE 1:**
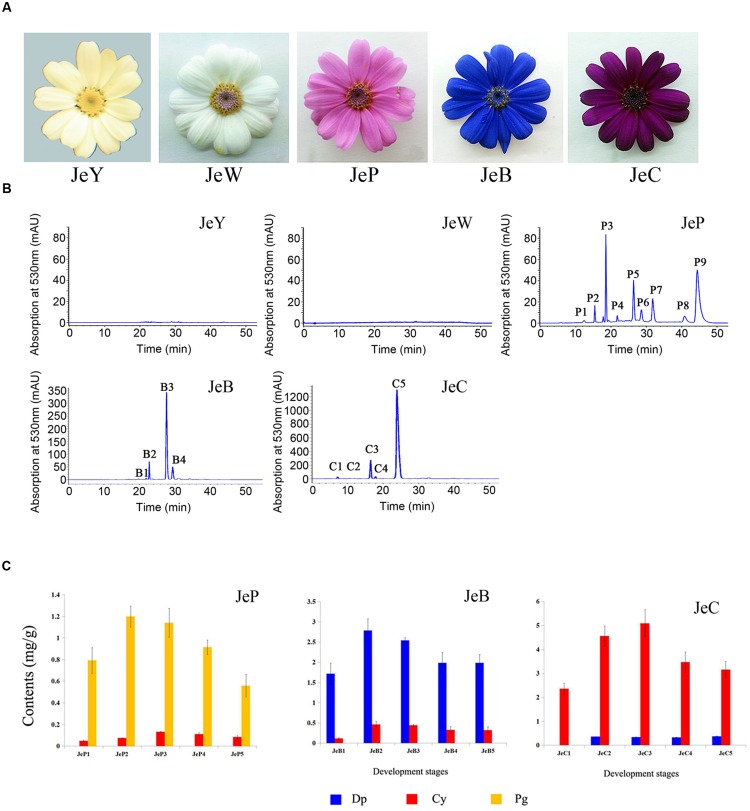
***Senecio cruentus* cultivars used in this study. (A)** JeY, JeW, JeP, JeB, and JeC. **(B)** Results of anthocyanins composition in different flower color cineraria cultivars by high performance liquid chromatography (HPLC) analysis. **(C)** Anthocyanins contents of JeB, JeC, and JeP determined by HPLC analysis.

*Senecio cruentus* ‘Jester’ was used, with its five flower colors, including white, yellow, pink, carmine and blue. The seeds of the five cultivars were introduces from Syngenta, USA. The yellow, white, pink, blue and carmine cultivars were named JeY, JeW, JeP, JeB, and JeC, respectively, in this study (**Figure 1A**).

There are five stages in the development of the flower capitulum: S1 was defined as when the ray florets were not yet out of bract (0–5 mm in ray flower length); S2 was defined as when the ray flowers were acicular and had barely outgrown the bract (5 mm in ray flower length); S3 was defined as when the ray flowers clearly outgrew the bract, but the capitulum was compact (5–8 mm in ray flower length); S4 was defined as when the ray flowers were opening and the angle between the ray flowers and the stem was more than 90° (12–18 mm in ray flower length); and S5, was defined as when there was a fully opened flower and the angle between the ray flowers and the stem was nearly 90° (16–20 mm in ray flower length). The ray flowers of these five developmental stages were collected under sterile conditions, quickly frozen in liquid nitrogen and stored at -80°C before the pigment content determination and RNA isolation.

### Flower Colorimeter Analysis, Anthocyanin Content Measurement, and HPLC Analysis

For an objective flower color evaluation, the color components of the CIE *L^∗^a^∗^b^∗^* coordinate, namely, lightness (*L^∗^*) and chroma [*c^∗^*: calculated as *c^∗^* = (a^∗2^ + b^∗2^)^1/2^], were measured with a hand spectrophotometer (NR-3000, Nippon Denshoku Industries, Co., Ltd, Tokyo, Japan). The lightness coefficient ‘a^∗^’ value represents greenish and redness as the value increases from negative to positive, and ‘b^∗^’ represents bluish and yellowish.

For the UV-Vis analysis, freeze dried tissue was used for the qualitative analysis of pigments. Samples of the ground freeze-dried petal tissue (50 mg DW) were initially extracted in 2 ml of petroleum ether and acetone (4:1) and 2 ml of methanol, acetic acid, water, and trifluoroacetic acid (70:3:27:1) for 24 h at 4°C in the dark, and the absorption spectrum (220–700 nm; 400–500 nm) was determined with the use of a spectrophotometer (TU-1901; Beijing Puxi, Co., Ltd).

For the measurement of the anthocyanin content of each cultivar, 0.25 g of the sample was ground into fine powder in liquid N_2_ and then homogenized in 1 ml of anthocyanin extracts [methanol:distilled water:methane acid:trifluoroacetic acid (70:27:2:1, v/v/v/v)] at 4°C for 24 h. After samples were centrifuged, the supernatants were filtered using medium-speed filter paper (Hangzhou Special Paper Industry, Hangzhou City, China), and the filtrate was the passed through a 0.22-μm reinforced nylon membrane filter (Shanghai ANPEL, Shanghai City, China) before subjecting it to high performance liquid chromatography (HPLC) analysis.

The HPLC system Dionex (Thermo Fisher Scientific, Inc., Sunnyvale, CA, USA), equipped with a P680 HPLC pump, UltiMate 3000 autosampler, Thermostatted Column Compartment -100 and Photodiode Array Detector -100, was used to separate the constituents of the ray floret extracts. A C18 ODS-80Ts QA column (150 mm × 4.6 mm I.D., Tokyo, Japan) was used. A 10-μl sample of each combined supernatant was quantified at a flow rate of 0.8 ml/min and a column temperature of 25°C. Using a linear gradient (53–30%) of Solvent A [distilled water:methane acid:trifluoroacetic acid (97.9:2:0.1, v/v/v)] in Solvent B [acetonitrile:methane acid:trifluoroacetic acid 62.9:35:2:0.1(v/v/v/v)], the resulting chromatograms were read at 530 nm for anthocyanins. For preparation of the standard solution, Cyanin chloride was accurately weighed and dissolved in methanol and then diluted to the appropriate concentrations. The quantitative analysis was based on the method described by Sun et al. (2009). Each sample run for HPLC was repeated three times under the same conditions.

### Measurements of the Intermediate Product

The ray florets of each flower color at S2 were sampled for flavonoid analysis using high-performance liquid chromatography-mass spectrometry/mass spectrometry (HPLC-MS/MS). Five-hundred milligrams of the sample was ground into a fine powder in liquid N_2_ and then homogenized in 5 ml of flavonoid extracts (methanol, MeOH) at 4°C for 24 h in darkness, with vortexing every 6 h. After samples were centrifuged, the supernatants were transferred to fresh tubes. Then, the mixture was passed through a 0.22-μm reinforced nylon membrane filter (Shanghai ANPEL, Shanghai City, China) before being subjected to HPLC-MS/MS analysis. A 10-μl sample of each combined supernatant was quantified by HPLC-MS/MS (Ultimate3000 – API 3200 Q TRAP) at a flow rate of 1 ml/min and a column temperature of 40°C. A 150^∗^4.6 mm column of MSLab HP-C18 and a linear gradient (95–0%) of Solvent A (water with 1 mmol/L ammonium acetate) in Solvent B (acetonitrile with 1 mmol/L ammonium acetate) were used for 5 min, followed by 100% Solvent B for 5 min. The mass spectrometry conditions were ion source: -ESI; scan mode: MRM; CUR: 20 psi; CAD: Medium; IS: -5000 V; TEM: 400°C; GS1: 55 psi; GS2: 60 psi; EP: -10; and CXP: -2.0. Standards of Nar, DHK, DHQ, DHM, Kae, Que and Myr were obtained from Sigma-Aldrich China (Shanghai). All standards were accurately weighed and dissolved in acetonitrile and then diluted to the appropriate concentrations. The mean values and SD were obtained from three biological replicates.

### Transcriptome Sequencing and Analyses

The total RNA of the S2 stage of JeB was extracted using the TRIzol (Invitrogen, Grand Island, NY, USA) method, and RNA quality was determined using a NanoDrop 2000. In total, 30 μg were used for the construction and sequencing of the libraries. Briefly, mRNA was purified from total RNA using oligo (dT) magnetic beads. The fragmentation buffer was added to break mRNA into shorter fragments. These short fragments were used as templates. Random hexamer primers were used to synthesize the first-strand cDNA. The second strand cDNA was synthesized using a buffer containing dNTPs, RNaseH and DNA polymerase I (Promega, Madison, WI, USA). Short fragments were purified with a QiaQuick PCR extraction kit (Qiagen, Hilden, Germany) and resolved using EB buffer for end reparation and adding poly (A). Then, the short fragments were connected with sequencing adapters. In addition, after agarose gel electrophoresis, the suitable fragments were selected as templates for PCR amplification and the library was constructed. Finally, the library was sequenced using an Illumina HiSeq TM 2000.

The *de novo* assembly of the transcriptome was performed with the short reads assembling program SOAP2 (Li et al., 2009). SOAP2 first combined reads of a certain length of overlap to form longer fragments without N, which are called contigs. Then, the reads were mapped back to the contigs with paired-end reads to detect contigs from the same transcript, as well as the distances between these contigs. Next, SOAP *de novo* connected the contigs using N to represent unknown sequences between each of the two contigs, and then, scaffolds were made. Paired-end reads were used again to fill any gaps in the scaffold to obtain sequences that had the least amount of Ns and that could not be extended on either end. Such sequences were defined as unigenes.

The assembled unigenes were annotated using the BLASTx alignment (E-value < 1 × 10^-5^) to protein databases, such as the National Center for Biotechnology Information non-redundant (NCBI nr) protein database^1^, the Swiss-Prot protein database^2^, the Kyoto Encyclopedia of Genes and Genomes (KEGG) pathway database^3^, and the Clusters of Orthologous Groups of proteins (COG) database^4^. The best-aligning results from the four databases were chosen to decide the sequence direction of the unigenes.

### Obtaining Gene Related to ABP and Phylogenetic Analyses

The key words “*CHI, CHS, F3H, F3′H, F3′5′H, DFR, ANS, FNS, FLS, MYB*, and *bHLH*” were used as queries to retrieve the corresponding unigenes in the library, and the search results were aligned by Blastx to obtain the 5*′* terminal fragment of the encoded genes.

For the *MYB* and *bHLH* genes, to study the candidate genes belonging to the various subfamilies of the gene family, the *MYB* and *bHLH* genes from *A. thaliana* and other model species were used to perform phylogenetic analyses. ClustalX was used to align these sequences, and MEGA4.0 was used to construct a circular phylogenetic tree. The genes were classified into the corresponding subfamily according to Hichri et al. (2011).

### Gene Expression, Hierarchical Analysis, and Statistical Analysis

For all of the ABP pathway structural genes, real-time quantitative reverse transcription-PCR (RT-qPCR) was used to analyze the expression pattern among the five cultivars (primer sequences were listed in Supplementary Table S1).

For the *MYB* and *bHLH* genes, because they both belonged to one large gene family, the gene expression patterns were first analyzed by RT-PCR. All of the primer sequences are described in Supplementary Table S1. To ensure accuracy, the expression patterns of candidate *MYB* and *bHLH* genes were further analyzed by qRT-PCR according to the method above. To gain a more global view of the transcriptional coordination of the genes involved in the biosynthesis of anthocyanin, hierarchical clustering analyses were further performed, the generated expression values were entered into the ClustalW software for expression pattern clustering, and TreeView was used to generate the expression pattern tree.

## Results

### Color Differences and Qualitative Analysis of Pigments

To evaluate petal colors, color differences were measured and presented on the *L^∗^–c^∗^* coordinate. *L^∗^* indicates the lightness of the color; specifically, low lightness contributes to a black appearance and high lightness contributes to a white appearance. The JeB and JeC that exhibited deeper colors showed lower *L^∗^* values, whereas the more pale cultivars JeP, JeW and JeY showed higher *L*^∗^ values (**Table 1**).

**Table 1:** Flower color *L^∗^*, *a^∗^*, *b^∗^*and *C* values in middle petals of *Senecio cruentus* of five colors at full opening stage.

Cultivar	*L^∗^* Value	*a^∗^* Value	*b^∗^* Value	*C* Value
JeY	88.73 ± 2.87	-9.92 ± 0.93	27.84 ± 1.96	29.55 ± 2.01
JeW	89.91 ± 2.64	-3.74 ± 0.90	7.14 ± 0.92	8.06 ± 1.49
JeP	50.00 ± 2.19	44.38 ± 1.44	-13.13 ± 0.59	46.28 ± 1.41
JeB	18.32 ± 1.22	42.93 ± 1.95	-52.92 ± 2.86	68.14 ± 2.01
JeC	16.71 ± 0.81	48.71 ± 3.17	-1.70 ± 0.77	48.74 ± 1.59

*The data is the average value ± standard error (N = 5); C value = (a^∗^^2^+b^∗^^2^)^1/2^.*

HCl-methanol extracts of each cultivar are shown in Supplementary Table S1. In the visible light region, JeB, JeC, and JeP had a maximum absorption wavelength of 530 nm, corresponding to anthocyanin pigments. In contrast, JeW and JeY had no detectable peak in the visible spectrum, indicating that no anthocyanins were accumulated in these cultivars. It was noted that the absorption was only presented between 220 and 400 nm or between 500 and 600 nm, indicating that anthocyanin and flavonoids, but not carotenoid, was present in the analytic samples.

### Major Classes of Color Compounds in the Five Cultivars by HPLC Analysis

High performance liquid chromatography analysis revealed that no anthocyanins or derivatives were detected in both JeW and JeY. For the other colored cultivars, anthocyanins were the main pigments. The structure of anthocyanidins in the colored cultivars have been identified previously (Sun et al., 2009; **Figure 1B**; **Table 2**). These three colored cultivars all contained two types of anthocyanin compounds that are responsible for color pigmentation: 26% of total flavonoids for Pg and 2% for Cy in JeP; 67% for Dp and 11% for Cy in JeB; and 92% for Cy and 1% for Dp in JeC, which means that Pg, Cy, and Dp are the main pigments in JeP, JeC and JeB, respectively.

**Table 2:** Structure identification of anthocyanidins in colored cultivars.

Cultivar	Ingredient	Retention time (min)	Mass spectrum result	Anthocyanidin
JeP	P1	12.1	455[M+H]^+^ (4.45), 433 (22.25), 271[Y0^+^] (100)	Pg
	P2	14.9	653[M+H]^+^ (7.47), 519(16.51), 271[Y0^+^] (100)	Pg
	P3	16.1	433[M+H]^+^ (0.49), 271[Y0^+^] (100)	Pg
	P4	18	519[M+H]^+^ (10.56), 433(7.61), 271[Y0^+^] (100)	Pg
	P5	22	843[M+H]^+^ (0.83), 519(13.32), 271[Y0^+^] (100)	Pg
	P6	23.7	703[M+H]^+^ (0.18), 519(0.60), 271[Y0^+^] (100)	Pg
	P7	24.7	859[M+H]^+^ (2.50), 611(7.76), 449(5.85), 287[Y0^+^] (100)	Cy
	P8	28.8	617[M+ Na]^+^ (1.16), 433(9.42), 271[Y0^+^] (100)	Pg
	P9	30	843[M+H]^+^ (0.47), 519(12.47), 271[Y0^+^] (100)	Pg
JeB	B1	18.4	875[M+H]^+^ (13.16), 627(76.95), 303[Y0^+^](100)	Dp
	B2	19.5	875[M+H]^+^ (33.74), 627(24.57), 303[Y0^+^](100)	Dp
	B3	23.7	1523[M+H]^+^ (33.74), 875(6.42), 627(24.37), 303[Y0^+^](100)	Dp
	B4	24.7	859[M+H]^+^ (3.46), 611(14.30), 449(6.24), 287[Y0^+^](100)	Cy
JeC	C1	14.2	859[M+H]^+^ (3.90), 611(5.32), 449(5.44), 287[Y0^+^] (100)	Cy
	C2	17.3	795[M+Na]^+^ (11.28), 611(4.53), 465(25.97), 303[Y0^+^](100)	Pg
	C3	20.5	859[M+H]^+^ (7.39), 611(7.33), 449(0.65), 287[Y0^+^] (100)	Cy
	C4	21.2	859[M+H]^+^ (5.18), 611(27.73), 449(1.49), 287[Y0^+^] (100)	Cy
	C5	24.7	859[M+H]^+^ (5.27), 611(19.52), 449(5.10), 287[Y0^+^] (100)	Cy

To correlate pigmentation formation with gene expression, we analyzed the anthocyanin accumulation in five developmental stages of the petals of *S. cruentus* in the colored cultivars. The results showed that the accumulation of distinct types of anthocyanins started at S2 in all of the cultivars (**Figure 1C**).

The intermediate products that were involved in the metabolic process and its main branches were also compared. In JeW, DHK, and NAR were the main compounds, accounting for 60 and 22% of the total flavonoids. In JeC and JeB, the amount of the three types of dihydroflavonol were very small, suggesting that the metabolic flux had flowed down to leucocyanidin and anthocyanin. It should be noted that in JeP, the content of DHK was very high and accounted for 60% of the total flavonoids, which was almost 100-fold higher than the levels in the other four cultivars. In all five cultivars, the total content of the other flavones, such as kaempferol, quercetin and myricetin, did not exceed 5% of the total flavonoids content (**Table 3**).

**Table 3:** The contents of flavonoids in flower petals of *S. cruentus*.

	Contents (mg g^-1^)				
Standard	JeY	JeW	JeP	JeB	JeC
Delphinidin	nd	nd	nd	2.785 ± 0.298	0.035 ± 0.007
Cyanidin	nd	nd	0.076 ± 0.015	0.459 ± 0.078	4.556 ± 0.426
Pelargonin	nd	nd	1.198 ± 0.092	nd	nd
Naringenin	0.159 ± 0.001	0.859 ± 0.002	0.023 ± 0.001	0.006 ± 0.000	0.008 ± 0.000
Apigenin	nd	nd	nd	nd	nd
Luteolin	0.015 ± 0.001	0.053 ± 0.001	nd	nd	nd
Myricetin	0.039 ± 0.001	0.057 ± 0.001	0.066 ± 0.001	0.292 ± 0.002	0.134 ± 0.001
Quercetin	0.023 ± 0.001	0.048 ± 0.001	0.064 ± 0.001	0.131 ± 0.002	0.094 ± 0.001
Kaempferol	nd	nd	0.009 ± 0.000	0.002 ± 0.000	nd
Dihydromyricetin	0.026 ± 0.001	0.017 ± 0.001	0.015 ± 0.001	0.038 ± 0.001	0.017 ± 0.001
Dihydroquercetin	0.004 ± 0.000	0.008 ± 0.000	0.156 ± 0.001	0.105 ± 0.002	0.055 ± 0.001
Dihydrokaempferol	0.004 ± 0.000	0.432 ± 0.002	3.00 ± 0.032	0.136 ± 0.002	0.05 ± 0.001

### RNA-Seq and Annotation of Unigenes

A total of 52,879,884 reads were obtained by RNA-seq, with total nucleotides of 4,759,189,560 bp (4.76 GB; SRA accession number: SRX1992289). After removing the low quality reads, the remaining reads were assembled by the SOAP *de novo* software and 57,818 unigenes with an average length of 708 bp were identified. To assess the quality of the unigenes in the transcriptome library of *S. cruentus*, we first analyzed the proportion of unigenes that contained a gap and found that 92.16% of the unigenes did not contain a gap. There were 13,544 unigenes between 500 and 1,000 bp and 13,433 unigenes larger than 1,000 bp, which accounted for 46.66% of the total unigenes (**Table 4**).

**Table 4:** Summary of the *S. cruentus* ray floret transcriptome.

Transcriptome quality	Summary

Statistics of sequencing	Total raw reads	57,976,400
	Total clean reads	52,879,884
	Total clean nucleotides (bp)	4,759,189,560
	Q20 percentage	97.85%
	GC percentage	44.43%
Assembly quality of contigs	Total number	135,484
	Total length (bp)	42,636,598
	Mean length (bp)	315
	N50	545
Assembly quality of unigenes	Total number	57,818
	Total length (bp)	40,938,088
	Mean length (bp)	708
	N50	1,074
	Total consensus sequences	57,818
	Distinct clusters	27,702
	Distinct singletons	30,116

The complete genome of *S. cruentus* has not yet been made available. Therefore, we used four public databases (nr, Swiss-Prot, KEGG, and COG) to annotate the unigenes that we identified. Blastx was used to search for the unigenes against the public databases with the priority order of Nr, Swiss-Prot, KEGG and COG, and a cut-off E-value that was above 1.00E^-5^ was used.

Using this approach, 75.32% of the total unigenes (57818) were annotated. There were 16,601 proteins that were longer than 300 nt. The remaining unigenes were predicted by the EST scan. In total, 2,537 unigenes were annotated, and of these, the length of 113 protein products was more than 300 nt.

**FIGURE 2:**
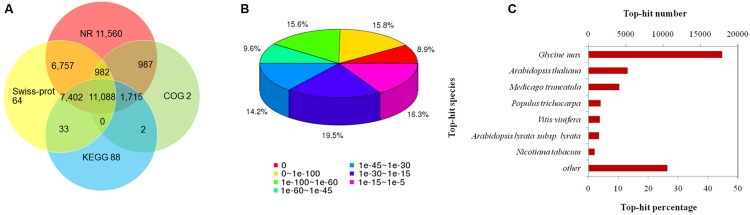
**Characteristics of the homology search of *S. cruentus* unigenes. (A)** Venn diagram of the number of unigenes annotated by BLASTx with an E-value threshold of 10-5 against four protein databases. **(B)** E-value distribution of the top BLASTx hits against the nr database for each unigene. **(C)** Numbers and percentages of unigenes matching the 20 top species using BLASTx in the nr database.

**FIGURE 3:**
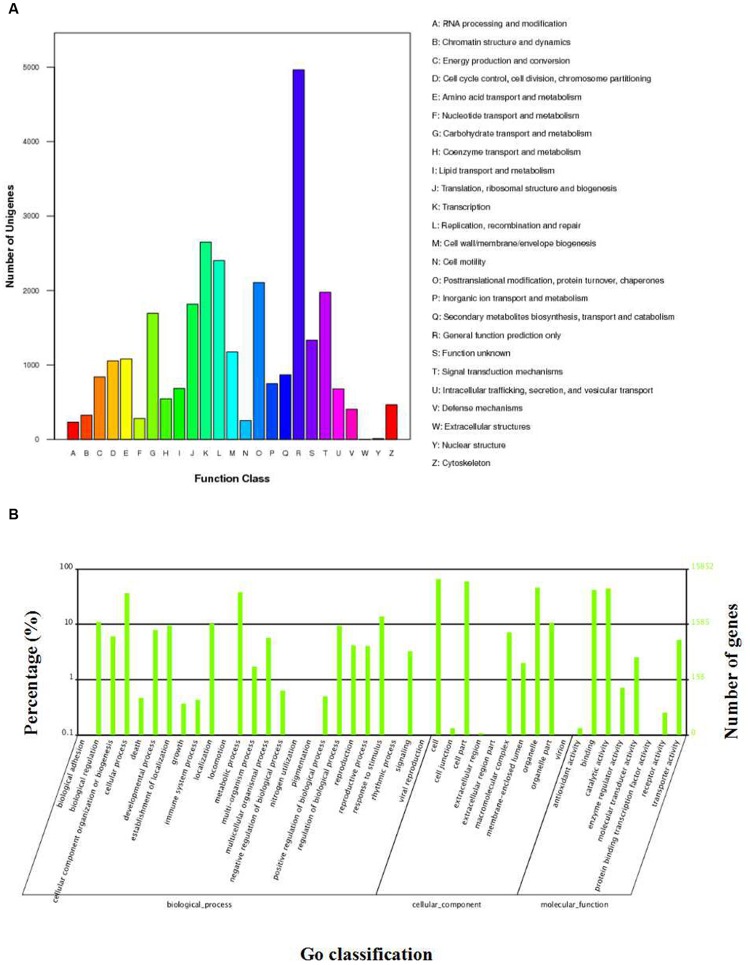
**GO and COG classification of *S. cruentus* unigenes. (A)** COG classification. **(B)** GO classification.

Among the annotated 57,818 unigenes, 43,367 could be annotated to the Nr database, which accounted for 75.0% of the total annotated unigenes (**Figure 2A**). Statistical analysis of the E-value characteristics distributed in the Nr annotation revealed that 83.64% of the mapped sequences showed strong homology (E-value < 1.00E^-15^) and 40.31% showed very strong homology (E-value < 1.00E^-60^) to the available plant sequences (**Figure 2B**). Based on the Nr annotation, 15,852 unigenes were classified into 43 functional categories using Blast2GO software, of which 25 GO terms were related to biochemical processes, 10 were related to cellular components, and 8 were related to molecular functions (**Figure 3A**; Supplementary Table S2). The distributions of the top 20 species for the best match from each sequence were shown in **Figure 2C**.

The three largest percentages of genes identified within these three functional categories were metabolic cells (10400, 65.61%), cell parts (9417, 59.41%), and organelles (7282, 45.94%). In addition, there were elevated percentages of genes involved in catalytic activity (molecular functions; 7037, 44.39%), binding (molecular functions; 6610, 41.70%) and metabolic processes (biochemical processes; 6037, 38.08%), whereas low percentages of genes (<0.02%) were related to the rhythmic process and nitrogen utilization (biochemical processes; **Figure 3A**).

To further demonstrate the potential functions of the annotated unigenes, 23,204 unigenes were mapped onto 125 KEGG pathways. The maps that had the highest number of unigenes represented were the metabolic pathways (4912 unigenes, 21.17%, ko01100), followed by the biosynthesis of secondary metabolites (2444 unigenes, 10.53%, ko01110). The maps that had higher numbers of unigenes represented were the genes that were involved in the plant-pathogen interaction pathway (1526 unigenes, 6.58%, ko04626), the spliceosome pathway (901 unigenes, 3.88%, ko03040), and the starch/sucrose metabolism pathway (567 unigenes, 2.44%, ko00500; Supplementary Table S3).

To investigate the phylogenetic lineages of the unigenes in the transcriptome library of *Cineraria*, we performed COG clustering analysis. The results revealed that 14,776 unigenes were divided into 25 categories (Supplementary Table S4). Among them, the largest group (group R, 4963, 33.59%) showed the predicted general functions, followed by the group with transcriptional functions (group K, 2650, 17.93%; **Figure 3B**). These data will be useful in the study of protein classification and evolution rate of *S. cruentus.*

### The Expression Patterns of the ABP Genes

All of the structural and regulatory genes were then isolated with the goal of determining the regulation of the anthocyanin biosynthesis genes in *S. cruentus*. Almost all of the genes belonged to known gene families, except for *F3′5 ′H*, *ANS* and *F3H*, which were single copy genes (Supplementary Table S5). For the MBW genes, a total of 27 *R2R3-MYB*, 18 *bHLH*, and 2 *WD40* genes were isolated. All of these genes were used for the gene expression analysis of S1–S5.

**FIGURE 4:**
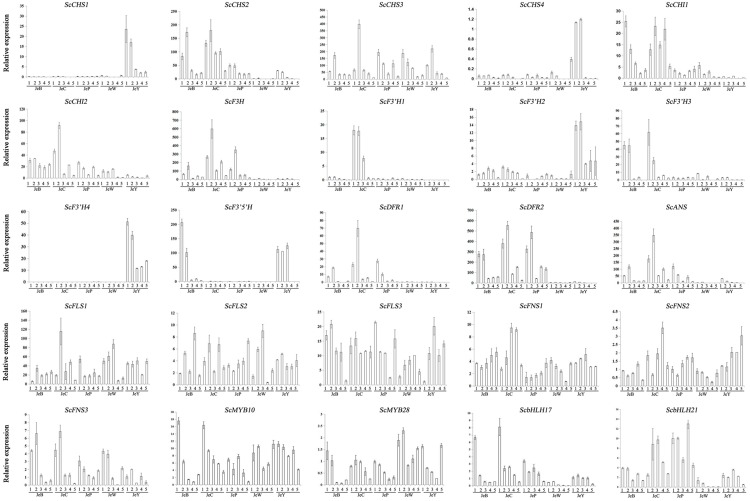
**Real-time quantitative reverse transcription-PCR (qRT-PCR) of ABP genes in *S. cruentus***.

In **Figure 4**, four members of the *CHS* gene family are shown. Among them, *ScCHS2* and *ScCHS3* were expressed in the three colored cultivars, and the expression patterns were positively correlated with the anthocyanin accumulation pattern (**Table 4**; Supplementary Table S3). *ScCHS1* and *ScCHS4* were only expressed in JeY. None of the *ScCHS* members were detected in JeW. Of the two *ScCHI* genes, both were expressed in the colored cultivars and JeW, whereas the expression of these genes at the transcriptional level was not detected in JeY. We found only one copy of *ScF3H* that was expressed in all of the colored cultivars, but with very low expression levels observed in JeW and JeY. A single copy of the gene *ScF3′5′H* was also detected, but was only expressed in JeB and JeY. There are four gene members of the *ScF3′H* family. Among them, *ScF3′H2* and *ScF3′H4* were highly expressed in JeY, whereas *F3′H1* was highly expressed in JeC, at a level that was 21-fold higher than in JeB. No *ScF3′H* genes were detected in JeW and JeP. In the case of the three late biosynthesis genes (LBGs), *ScDFR1*, *ScDFR2* and *ScANS*, all showed similar expression patterns that positively correlated with the anthocyanin accumulation pattern and all were first increased at the S2 and S3 and then decreased (**Table 3**; Supplementary Table S3). Meanwhile, these three genes were detected at the transcriptional level only in the colored cultivars. In the case of the flavonol and flavone biosynthesis genes, *ScFLS* and *ScFNS*, all of the members showed no specific anthocyanin type or accumulation related to the gene expression pattern among the five cultivars (**Figure 4**).

**FIGURE 5:**
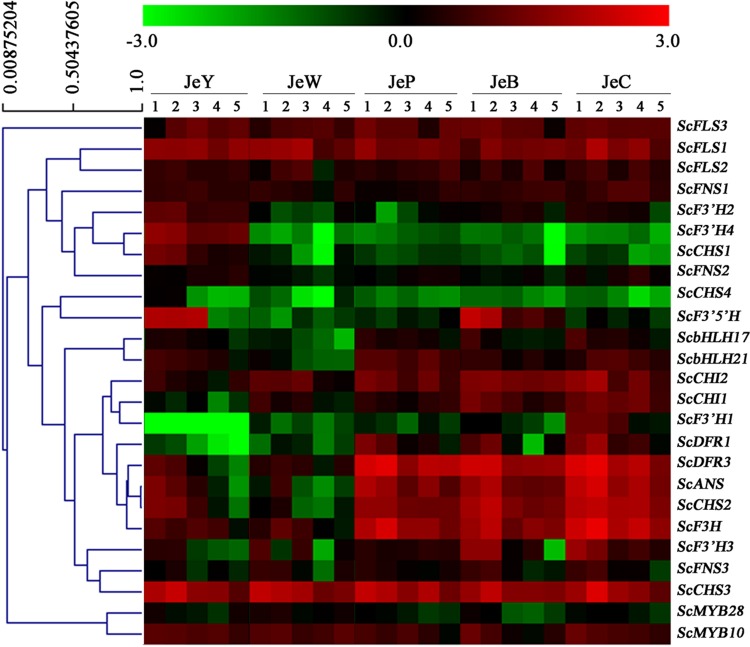
**Phylogenies analysis and RT-PCR of R2R3-MYB and bHLH genes in *S. cruentus***.

The expression patterns of the anthocyanin regulatory genes, including *MYB* and *bHLH*, were also investigated by RT-PCR and qRT-PCR. All of the *MYB* genes were constitutively expressed in the five cultivars. For the *bHLH* genes, *ScbHLH17* was expressed in the three colored cultivars and the expression pattern correlated perfectly with the anthocyanin accumulation profiles among the cultivars (**Figure 5**).

**FIGURE 6:**
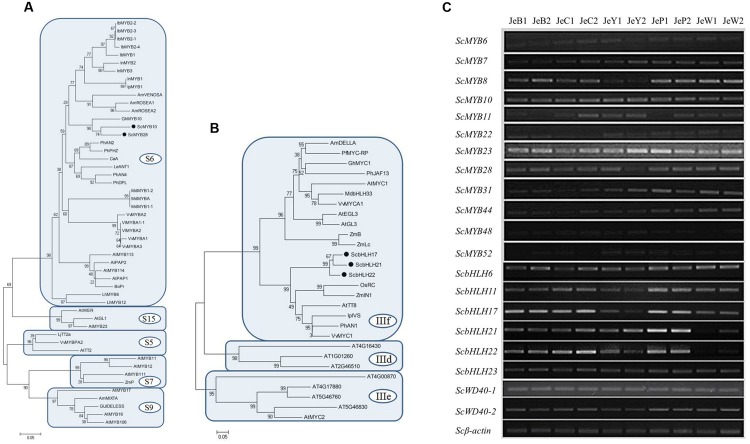
**Hierarchical clustering analysis of the expression patterns of anthocyanin genes in *S. cruentus.* (A)** Phylogenetic relationship among *ScMYBs* genes and other plant *MYB* homologous genes. **(B)** Phylogenetic relationship among *ScbHLHs* genes and other plant *bHLH* homologous genes. **(C)** Expression patterns of all *S. cruentus* regulatory genes among five cultivars.

To gain a global view of the transcriptional coordination of the genes involved in anthocyanin biosynthesis, hierarchical clustering analysis was performed. As shown in **Figure 6**, *ScCHS2, ScCHI1, ScCHI2, ScF3H*, *ScF3′H1*, *ScDFR1, ScDFR3*, *ScANS* and the transcription factor genes *bHLH17, ScbHLH21* were clustered together, suggesting that there is co-expression over the five development stages.

## Discussion

In this study, through a combination of chemical and transcriptomic analyses, the major metabolic pathways related to different cultivars of *S. cruentus* flower pigmentation were investigated, the candidate genes that determined the biosynthesis and branch were isolated, and the regulatory mechanism of key genes was discussed.

### Reasons for the Loss of Anthocyanin in the White and Yellow Cultivars

Both JeY and JeW cannot accumulate anthocyanins. However, the underlying mechanisms were different between them. In JeW, the ABP pathway is blocked and the expression levels of most of the genes in the ABP pathway are inhibited, including *CHI*, *F3H*, *F3′H*, *DFR* and *ANS*, but not *CHS*, the expression level of which is not inhibited. All of these results suggest that the inhibition of the expression of regulatory genes may be the key reason underlying the absence of expression of the structural genes. The mutation of genes encoding transcription factors that cause white flower formation have been reported in many species (Espley et al., 2009; Chiu et al., 2010). Correlation analysis and clustering analysis have also shown that the expression level of the structural genes correlates with that of *ScbHLH17*, a gene that is highly expressed in all of the other four colors but that shows low expression levels in JeW.

In theory, shifts from pigmented to white flowers could involve any mutations that block one or more of the steps in the anthocyanin pathway. These include loss-of-function (LOF) mutations of any pathway enzyme, including the LOF of any of the three proteins (R2R3-MYB, bHLH, and WD40) that regulate the expression of the enzyme-coding genes as well as the *cis*-regulatory mutations that downregulate any of the pathway enzymes. Characterization of spontaneous white flower mutants in *Petunia*, *Antirrhinum*, and *Ipomoea*, as well as in other ornamental plants, has demonstrated that there are roughly equal frequencies of both regulatory and functional mutations. Specifically, 29 of the 69 tabulated cases involve spontaneous functional mutations of core pathway enzymes, 8 of the 69 cases involved spontaneous *cis*-regulatory mutations of core pathways enzymes, and 32 of the 69 cases involved spontaneous transcription factor mutations (Wessinger and Rausher, 2012).

From the *L^∗^* value, we can see that the flower color of JeY was, and can only be regarded as, a pale yellow color. The results from the UV-Vis analysis also showed that there is no accumulation of carotenoids in JeY. The expression analysis of the structural genes showed a high level of expression of *CHS* and a low level of expression of *CHIs*, suggesting that a large amount of chalcones accumulate and cannot move downstream. Although *F3H*, *DFR*, *ANS*, and *F3′5′H* can be highly expressed, the lack of a substrate renders them unable them to function. All of these results have proven that *CHI* may play a critical role in the coloration of pale yellow flowers, which is in agreement with previous reports. For example, Nishihara et al. (2005) used RNAi and transgenics to inhibit the expression of *CHI* in *Nicotiana tabacum* (tobacco), which resulted in a reduced accumulation of anthocyanin and a higher chalcone content that eventually led to yellow coloration (Nishihara et al., 2005). *Ipomoea chi* mutants have been shown to produce pale yellow flowers that accumulate chalcone 2′-*O*-glucoside rather than lacking flavonoids (Morita et al., 2014). The yellow seeds of *Arabidopsis chi* mutants accumulate chalcone derivatives and have reduced amounts of flavonols (Böttcher et al., 2008).

### The Relationship between the Anthocyanin Synthesis Pathway and the Flavone and Flavonol Pathways in *S. cruentus*

The relationship between the anthocyanin synthesis pathway and the flavone and flavonol pathways has been studied in many species, and it has been suggested that they are in competition. Specifically, in plants, blocking the accumulation of anthocyanin strengthens the metabolic flux toward the flavonols (Davies et al., 2003; Zhang et al., 2014).

The actions of the FNS and the flavonol synthase (FLS) lead to the branching of the flavonoid pathway to flavones and flavonols, respectively (Tanaka and Brugliera, 2013). This competition has been reported in many species. Previous studies have indicated that the metabolic flux in the flavonoid biosynthetic pathway is controlled by substrate competition between the FLS and the DFR in *Arabidopsis* (Gou et al., 2011). In Dahlia, a study comparing the black cultivar ‘Kokucho’ and its purple mutant has also shown that the suppression of *DvFNS* abolishes the competition between anthocyanidin and flavone synthesis and leads to the accumulation of Cy and total anthocyanidins that produce a black appearance (Deguchi et al., 2013). In *M. armeniacum*, alteration of the substrate competition between the FLS and the DFR may lead to the elimination of blue pigmentation, while the multishunt from the limited flux in the Cy synthesis pathway seems to be the most likely reason for the color change in the white flowers (Lou et al., 2014). The overexpression of *Epimedium sagittatum EsFLS* in tobacco has resulted in increased flavonol content and decreased anthocyanin content in flowers (Huang et al., 2015).

In *S. cruentus*, we did not observe the competition between the anthocyanin synthesis pathway and the flavone and flavonol pathways because there was no significant difference in either the amount of all of the flavones and flavonols or the expression of the *FLS* and *FNS* genes in the five cultivars. Furthermore, the flavones and the flavonols are also important accessory pigments. The accessory pigments function when the co-pigmentation index (CI) is larger than 5 (Asen et al., 1971). Our previous study has shown that the CI is less than 3 in all of the cultivars of *S. cruentus*, which suggests that the flavones and the flavonols have little effect on the coloration of *S. cruentus* (Sun et al., 2009).

### In *S. cruentus*, *F3′5′H* and *F3′H* Play Important Roles in the Accumulation of Dp and Cy, Respectively

Both JeC and JeB can accumulate two different types of anthocyanin pigments, Cy and Dp. However, the content of Dp in JeB was much higher than the content of Cy, while in JeC, the opposite was true. The pigment flow analysis showed that the contents of Nar, DHK and other flavonoids were basically identical between the blue and carmine flowers. This is not the case with DHK, the branching point of anthocyanin biosynthesis. In JeB, approximately 75% of DHK went downstream to DHM, ultimately producing Dp, and 21% flowed downstream to the DHQ, ultimately producing Cy, while in JeC, almost 95% of DHK were synthesized to DHQ and only approximately 4% went downstream to DHM, ultimately producing Dp.

The gene expression analysis showed that JeB and JeC showed no difference in their expression of *CHS*, *CHI*, *F3H*, *DFR* and *ANS*, or in the regulatory genes. However, in JeB, *F3′5′H* and *F3′H3* were highly expressed, and these genes catalyze a large portion of the DHK to the Dp branch and a smaller portion to the Cy branch, respectively. While in JeC, the expression level of *F3′5′H* was very low, whereas the *F3′H1* and *F3′H3* were highly expressed, causing DHK to move downstream toward the Cy branch. Therefore, we believe that the ratio of expression levels of *F3′H* and *F3′*5*′*H showed strong correlations with the metabolic flow direction at the DHK branching point.

Various flower color mutations have been described in many plant species. Mutations in the *F3′H* or *F3′5′H* genes result in significant flower color changes. It has been documented that the ratio of *F3′5′H/F3′H* transcription controls the composition and proportion of flavonoids detected in different tissues and cultivars (Smith et al., 2013). In *Vitis vinifera* (grape berries), violet/blue cultivars produce more Dp derivatives and have a higher ratio of *F3′5′H*/*F3′H* transcription than the red cultivars (Castellarin et al., 2006). In *Lycium ruthenicum*, the difference in the bioactivity of the enzymes encoded by F3′H and F3′5′H likely plays a decisive role in directing the Cy and Dp flux (Zeng et al., 2014).

Some reports have shown that the F3′5′H in Compositae belongs to the CYP75B rather than to the CYP75A subfamily, in which the species lose a CYP75A type F3′5′H gene and then reacquire the F3′5′H gene by duplication and neofunctionalization of a CYP75B gene (Seitz et al., 2006). By expressing genes in the *Petunia* that were deficient in the *F3*′*H* and *F3*′*5*′*H* genes, Tanaka reported that the CYP75 members from cineraria are F3′H and F3′5′H (Tanaka and Brugliera, 2013). However, in accordance with our previous studies, the results from our gene expression and HPLC analysis show that the content of Dp was much higher than Cy in JeB, suggesting that there is a very high CYP75A activity of *ScF3*′*5*′*H* (Huang et al., 2013; Sun et al., 2014).

### The *DFRs* Play Important Roles in the Accumulation of Pg in *S. cruentus*

The DFR is known to catalyze the reaction that transforms dihydroflavonols (DHK, DHQ, and DHM) to leucoanthocyanidins (LPg, LCy, and LDp) using β-NADPH. Two variants of DFRs have been reported previously in the literature: non-specific DFRs that convert all of the types of dihydroflavonols and specific DFRs that convert only DHQ and DHM (Miosic et al., 2014). *ScDFR1* and *ScDFR2* could be expressed in all three colored cultivars, and their expression patterns were positively correlated with the accumulation of anthocyanin, suggesting that both of these DFRs belonged to the non-specific DFR that is able to convert all types of dihydroflavonols. We speculated that this was the reason why *S. cruentus* could accumulate all three types of anthocyanins, but the causalities between the gene expressions and metabolic flow direction still needed further study.

In JeP, both *F3*′*H* and *F3*′*5*′*H* were not expressed, whereas, two members of the DFR family showed higher expression levels. The pigment analysis indicated that there was no accumulation of Cy, Dp, DHM, and DHK in the pink flower, but the content of DHQ was high, approximately 30–100 times higher than other colors. From these results, we believed that the low expression of *F3*′*H* and *F3*′*5*′*H* coupled with the high expression of *DFRs* can catalyze the DHK that makes the anthocyanin biosynthesis flow to the Pg branch, eventually leading the flowers to display a pink color. Similar reports have demonstrated that DFR is able to play a catalytic role in the formation of Pg only when both F3′5′H and F3′H lose their catalytic activities. In *I. quamoclit*, a mutation in the coding region of the *F3′H* gene results in its inability to produce Cy-derived anthocyanins (Zufall and Rausher, 2004). Flux is then redirected from the Cy branch of the biosynthetic pathway to the Pg branch, thereby producing red flowers. In *Torenia fournieri*, cyclamen and gentian, Pg production was achieved by the suppression of the endogenous *F3′5′H* and *F3′H* genes and the expression of DFR with appropriate substrate specificity (Tanaka and Brugliera, 2013).

## Conclusion

**FIGURE 7:**
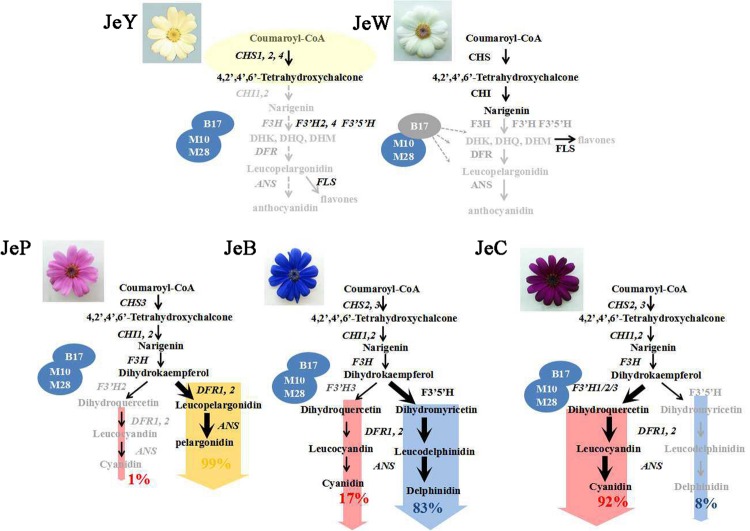
**Conclusive pathways for *S. cruentus* flower expressions**.

In summary, we have demonstrated the coloration mechanism underlying the different colors of *S. cruentus* (**Figure 7**). Due to the low expression level of the transcription factor *ScbHLH17*, the expression levels of structural genes, such as *ScCHS3*, *ScF3H1*, *ScDFR1*, *ScDFR3*, *ScANS*, were very low, which caused no formation of anthocyanins in JeW. However, in JeY, although the structural genes in the ABP pathways and the transcription factors were expressed, two members of the *CHI* family were not expressed, resulting in the inability of chalcone to be catalyzed to naringenin, leading to a pale yellow flower color. The difference in the coloration mechanisms among the three colored flowers was derived from the DHK branching point. In JeB, *F3′5′H* was overexpressed, whereas only one member of the *F3′H* family had a low expression level contributing to the accumulation of Dp. In JeC, *F3′5′H* had a low expression level and two members of *F3′H*, *ScF3′H1*, and *ScF3′H3* were highly expressed in the metabolic flux, resulting in flow to the Cy branch. In JeP, both *ScF3′5′H* and *ScF3′Hs* were unable to be expressed. Therefore, the two non-specific DFRs used DHK as their substrate causing the pink flower to accumulate Pg.

For many years, plant breeders have sought to generate novel flower colors in popular ornamental species. Transgenic approaches have become necessary in the absence of the necessary natural genetic variation in the target species. The anthocyanin branching point genes, including *F3′5′H*, *F3′H* and *DFRs*, are all crucial gene resources for the future transgenic breeding of ornamental plants.

## Author Contributions

The study was conceived by SD and HH. XJ performed the transcriptome and gene expression analysis. HH and LW conducted bioinformatics analyses and data interpretation. LW, XJ, and YS carried out the metabolite analysis experience. SD and HH participated in the preparation of the manuscript. All authors contributed to revising the manuscript. All authors had read and approved the final manuscript.

## Conflict of Interest Statement

The authors declare that the research was conducted in the absence of any commercial or financial relationships that could be construed as a potential conflict of interest.

## Footnotes

1 http://www.ncbi.nlm.nih.gov

2 http://www.expasy.ch/sprot

3 http://www.genome.jp/kegg

4 http://www.ncbi.nlm.nih.gov/COG
